# Assessing the readiness of health facilities to provide family planning services in low-resource settings: Insights from nationally representative service provision assessment surveys in 10 Countries

**DOI:** 10.1371/journal.pone.0290094

**Published:** 2023-11-16

**Authors:** Mosiur Rahman, Md. Jahirul Islam, Izzeldin Fadl Adam, Nguyen Huu Chau Duc, Prosannajid Sarkar, Md. Nuruzzaman Haque, Md. Golam Mostofa

**Affiliations:** 1 Department of Population Science and Human Resource Development University of Rajshahi, Rajshahi, Bangladesh; 2 Griffith Criminology Institute, Griffith University, Mount Gravatt, Queensland, Australia; 3 Faculty of Public Health, University of Khartoum, Khartoum, Sudan; 4 Hue University of Medicine and Pharmacy, Hue University, Hue, Vietnam; 5 Dr. Wazed Research and Training Institute, Begum Rokeya University, Rangpur, Bangladesh; Johns Hopkins Bloomberg School of Public Health, UNITED STATES

## Abstract

**Background:**

Many low-income countries continue to have high fertility levels and unmet need for family planning (FP) despite progress in increasing access to modern contraceptive methods and in reducing the total fertility rate (TFR). Health facilities in sub-Saharan Africa (SSA) and South Asia (SA) are thought to be unable to adequately deal with the burden of high unmet FP demands due to their weaker health systems. As a result, determining the readiness of health facilities that offer FP services is critical for identifying weaknesses and opportunities for continued development of FP health systems in those regions. Service Provision Assessment (SPA) tools—which break down health systems into measurable, trackable components—are one useful way to assess service readiness and the ability of health institutions to deliver FP services.

**Methods:**

Using data from nationally representative SPA surveys, we conducted a study that aimed to: (1) evaluate healthcare facilities’ readiness to provide FP services; and (2) identify the factors that affect FP service readiness. Using a cross-sectional survey design, we used data from SPA surveys conducted in 10 low-resource SA and SSA countries: Afghanistan, Bangladesh, Kenya, Malawi, Namibia, Nepal, Rwanda, Senegal, Tanzania, and the Democratic Republic of the Congo (DRC). We analyzed data from public and private health facilities in Afghanistan (84), Bangladesh (1,303), Kenya (567), Malawi (810), Namibia (357), Nepal (899), Rwanda (382), Senegal (334), Tanzania (933), and the DRC (1,061) for a total of 6,730 facilities. We used 17 items/indicators recommended by the Service Availability and Readiness Assessment to measure a health facility’s readiness to provide FP services across four domains.

**Results:**

Only 3.6% to 34.1% of the health facilities were reporting at least 75% (12–13 of 17) of the relevant items for FP service provision. Most of the health facilities in the countries under investigation suffered from lack of readiness, meaning that they did not fulfill at least 75% of the standards (12–13 items of 17 items on the availability of trained staff and guidelines, equipment, and commodities components). The factors associated with higher readiness scores varied among the 10 countries analyzed. Regression models showed that increases in the number of FP healthcare providers available at a health facility and infection control measures for FP exams were factors linked to increased readiness scores in all 10 countries. The low readiness of health facilities to provide FP services in the countries studied showed that the health systems in these low-resource settings faced significant problems with providing FP services. Differences in country-specific variability in the characteristics linked with better preparedness ratings could be attributed to data collected across different years in different nations or to country-specific healthcare financing policies.

**Conclusions:**

To increase a health facility’s readiness to offer FP services, country-specific factors must be addressed, in addition to common factors found in all 10 countries. Further research is required to determine the causes of country-level differences in FP tracer item availability to develop targeted and effective country-specific strategies to improve the quality of FP services in the SA and SSA regions and address unmet need for FP.

## Introduction

Rapid population growth continues to be a major concern for many low- and middle-income countries (LMICs), especially those in South Asia (SA) and sub-Saharan Africa (SSA), due to higher total fertility rate (TFR) and maternal mortality rate (MMR) than the global average. This is true even though there has been significant progress over the past decade in reducing the TFR and the MMR [1]. In 2017, about 86% of the reported global maternal deaths were in SSA and SA [1]. Family planning (FP) is one of the most cost-effective interventions to improve mother and child health outcomes. It helps women and couples achieve their desired fertility by preventing unintended and undesired pregnancies, which slows the growth of the population and lowers maternal and neonatal mortality [2]. This led many LMICs in the 1960s to launch programs to encourage the use of FP [3]. From 1960 to 1996, the number of LMICs providing FP services grew from two to 115 [4]. Consequently, between the 1960s and 1996, the TFR dropped from 6.1 to 3.3 in the LMICs [4].

Although progress has been made in increasing access to modern FP methods, many LMICs continue to have higher unmet FP needs. Demographic and Health Surveys (DHS) conducted in 52 LMICs between 2005 and 2014 found that an estimated 225 million women had an unmet need for contraception [5]. Although SSA has the greatest proportion of women with unmet FP needs (24%), it is still relatively high in SA (approximately 14%) [5]. Enhancing health facilities’ readiness for providing FP services such staff and guidelines, equipment and supplies, medicines, and commodities, is one method for boosting contraceptive uptake and decreasing unmet need.

Due to their weaker health systems, health facilities in SSA and SA are considered to be inadequately equipped to handle the burden of increased unmet FP demands. For instance, whereas 98% of Nepal’s health facilities offer at least one FP method, the readiness index’s constituents showed that just 46% and 39% of the facilities, respectively, possessed FP staff and guidelines [6]. In multicounty research, only 83% and 33% of health facilities in Tanzania and Democratic Republic of the Congo (DRC), respectively, offered general FP service [7]. As a result, determining the readiness of health facilities that offer FP services is critical to identify weaknesses and opportunities for continued development of FP health systems in those regions. Such information is needed to inform policymakers on how to improve health systems and to lessen the increased burden of unmet FP needs. Service Provision Assessment (SPA) tools, which break down health systems into measurable, trackable components, are one useful way to assess service readiness and the ability of health facilities to deliver FP services.

### Measuring health facility readiness for FP service provision

A number of tools, including COPE^®^ (Client-Oriented, Provider-Efficient) [8], Evaluation of Long Acting and Permanent Methods Services (ELMS) [9], Quick Investigation of Quality (QIQ) [10], Service Availability and Readiness Assessment (SARA) [11], and SPA [12], have been developed to assess a health facility’s readiness to offer FP services. However, aside from SPA, the majority of tools—including COPE, ELMS, and QIQ—were not designed for comparisons within or among healthcare facilities, lack publicly accessible data sets, and offer an insufficient number of tracer items to adequately capture various FP service delivery components. SPA is the most widely utilized, standardized method for assessing health facility readiness and yielding data that are nationally representative in LMICs.

By using SPA survey tools, detailed subnational and national assessments of health facility general service readiness [13, 14] and readiness for disease-specific studies [15–21] have been conducted to date. However, data on the ability and readiness of facilities to provide FP services are still scarce. Low readiness to provide FP services was found in a few studies conducted in Bangladesh [22], Kenya [23], Mozambique [24], Pakistan [25], Tanzania [26], and Uganda [27]. The majority of this research on the readiness of FP services in LMICs [22, 24, 25, 27], however, relied on small sample sizes, non-representative samples, or did not use a standard measurement instrument to assess readiness, which might bias the findings. Additionally, previous national survey research [23, 26], on this subject was primarily conducted in a single nation. Multi-country studies are required to present facility capacities to perform FP services for nations with comparable sociocultural traits in a way that is comparative.

There is only one known multi-country study that appears to have been done to assess how ready health facilities are to provide FP services [7]. But the data used for this study, which was based on the SARA survey, only included African nations. Though the SPA and SARA questionnaires both include a facility inventory module that gathers data on the general and specific service availability and readiness, evidence suggests that there are significant differences in the metrics and methods used to measure the quality of care between the two survey tools [28]. As a result, utilizing the SPA survey instruments, a multi-country evaluation is required to determine the level of readiness of health facilities for FP services.

### Study objectives

The study’s objectives were to: (1) assess the state of FP service readiness in 10 low-resource SA and SSA countries and (2) determine the factors associated with FP service readiness in the countries studied.

## Methods

### Data sources

This analysis utilized publicly available data sets from the cross-sectional Demographic and Health Surveys Program SPA of 10 countries: Afghanistan [29], Bangladesh [30], Kenya [31], Malawi [32], Namibia [33], Nepal [34], Rwanda [35], Senegal [36], Tanzania [37], and the DRC [38]. A detailed description for obtaining access and permission to analyze the DHS data is available (https://dhsprogramcom/data/Using-DataSets-for-Analysiscfm). When choosing these 10 countries using the SPA survey, the following criteria were taken into account: i) the existence of FP-related questions; ii) the availability of specific tracer items for FP services; and iii) the most recent survey, which was carried out within the previous decade between 2007 and 2019 (For details of the sample selection of the facilities included in the SPA sample, see S1 Fig).

Standardized facility inventories and questions for health care providers were used in SPA surveys [29–38]. Interviewers consulted the most knowledgeable person regarding service readiness both generally and for particular services like FP. Since our objectives were to examine the facilities’ readiness to deliver FP services rather than the credentials, training, and experience of healthcare providers, thus, only information from the facility inventory questionnaire was employed in this investigation.

The questionnaires for the SPA surveys were written in English and were translated into each country’s native language. The basic SPA questions of the DHS Program were fitted and customized to the country’s situation and demands. Countries utilizing the SPA chose 1 of 2 sampling methods: (1) a nationally representative simple/systematic random sample of health facilities—to produce national estimates, or (2) a census of all facilities in in selected districts, which can be utilized, if desired, for subnational estimates (For further information on the survey’s details, see S1 Table).

### Measures

#### Outcome variable

The outcome variable in this study was the health facility’s readiness to provide FP services. This was a composite measure calculated as a counting score based on the number of important indicators needed for FP services at a facility. The SARA reference manual [11] recommended three categories for evaluating readiness for service provision. Within these three categories, a total of 17 items/indicators were identified: (1) staff and guideline components (2 indicators); (2) equipment and supplies components (6 indicators); and (3) commodities components (9 indicators).

The availability of FP guidelines and at least one staff person who had ever received in-service FP training were used to evaluate the staff and guidelines domain. The presence of a blood pressure (BP) apparatus, examination light, examination bed, samples of FP methods, a model for displaying condom use, and visual aids were used to evaluate the second domain, the components of equipment and supplies for FP services. The third and final area, commodities, was assessed based on the facilities that provided clients with nine different types of modern FP methods: combination oral pill, progestin-only oral pill, progestin-only injectable, male condom, intrauterine device (IUD), implant, tubal ligation, vasectomy, and emergency contraception. Although sterilization was not listed in SARA [11] as a service commodity, this study considered vasectomy and tubal ligation as service commodities because sterilization is the most cost-effective form of contraception that is intended to be non-reversible.

Binary variables, such as presence or absence, were used to determine the availability of each of the 17 indicators. The readiness score was then summed up by adding the existence of each indicator (observed and seen by the interviewers). The contribution of each indicator to the overall score was given equal weight. The resulting readiness score, which was derived as a counting score based on the availability of the 17 SARA indicators, was used as the outcome variable. The scale ran from 0 to 17. Overall facility readiness was assessed as high if the health facility fulfilled standards (met at least 75% availability of the 17 items) among the three domains.

#### Explanatory variables

The explanatory variables were chosen based on two criteria: (1) the existence in the SPA survey survey’s data set of the countries under examination [29–38] and (2) prior research [23, 24, 26], which identified them as significant predictors of health facilities’ readiness to deliver FP services. The following variables were considered: managing authority; external sources of revenue; quality assurance activities; routine management meetings; external supervision; user fees; presence of a trained health provider at the facility 24 hours a day, on a duty schedule, or on-call; feedback on clients’ opinions; number of days that FP services were offered in a month; number of trained FP service providers; and the presence of infection prevention and control items in areas where FP services were offered.

A dichotomous variable was created to assess the managing authority (public facilities being owned by the government, or private/nongovernmental organization [NGO] being facilities not owned by the government). External revenue sources were divided into three categories based on whether the facilities received additional (extra) funding from the government, NGOs, or none at all. To track quality assurance actions, a dichotomous variable was created (yes: facility reported to routinely carry out quality assurance activities, [e.g., review of mortality, or audit of registers within the past 12 months], or no: facility did not report to routinely carry out quality assurance activities).

External supervision was marked as “yes” if the facility had received supportive supervision from a higher authority in the past six months, such as the district or regional health management team; otherwise, the facility was coded as “no.” A dichotomous variable was used to assess the frequency of routine management meetings (performed: whether the facility reported conducting regular management meetings at least once every 2–3 months, or not performed). There was also a binary variable added to define user fees as “none” or a “flat fee for all services.” The presence of a trained health professional at the facility 24 hours a day, on a duty schedule, or on-call was classified as “yes” or “no.”

The client feedback system was divided into two categories: reviewed and unreviewed. Seven standards for FP infection control measures, such as running water and soap or hand disinfectant, latex gloves, a sharps container, alcohol-based hand rub, and a trash receptacle, were all evaluated. The indicators were recorded as “yes” if the interviewers spotted the item and “no” if they were missing. The readiness score was then summed up by adding the existence of each indicator. Scores range from zero to seven. The number of FP suppliers was a discrete quantitative variable in this study.

### Statistical analyses

To begin, descriptive analyses were run to obtain general information about the sample’s characteristics. The mean was utilized in the descriptive analysis to summarize continuous variables. All categorical variables were summarized using proportions, which were then described using tables and graphs. The Chi-square test was used to compare the 17 components needed for health facilities to be ready to provide FP services across the countries.

The association between each selected explanatory variable and a facility’s readiness to provide FP was estimated using a negative binomial regression model because our outcome variable for the investigated countries was a count variable with overdispersion. We generated incidence rate ratios (IRR) using a negative binomial regression model. At the same time, all explanatory factors were put into the multiple regression models. The significance of the association was confirmed using a *P* value of <0.05 and a 95% confidence interval (CI) for the IRR. To account for non-response and disproportionate sampling, all estimates were weighted. We did not combine the data sets in our study; rather, we looked at each one separately; therefore, the weights of the pooled data were not de-normalized. Stata V.16 was used to analyze the data (StataCorp).

### Ethical considerations

The findings were based on a review of existing public domain survey databases, which were freely accessible online and provided disconnected information for all identifiers. The SPA survey was approved by the ethical review boards of each country’s MOH and ICF International’s institutional review board in the United States. Informed consent was obtained from the management, the facility’s person-in-charge, or the most senior health professional who was present at the facility and responsible for client services in all the sampled countries’ SPA surveys. Analyses of these data were excused from the Institute of Biological Sciences’ ethical review board (No. 293(13)/320/IAMEBBC/IBSc), University of Rajshahi, Bangladesh, due to the database’s anonymity. All study procedures were carried out in conformity with the principles of the 2013 revision of the Declaration of Helsinki.

## Results

### Characteristics of surveyed facilities

Table 1 presents the distribution of surveyed facilities depending on their background characteristics. In Bangladesh, Kenya, Malawi, Namibia, Nepal, Rwanda, Senegal, and the DRC, most facilities were public, whereas in Afghanistan and Tanzania, most were private/NGO. Most facilities in most countries surveyed reported receiving funds from government sources, whereas most facilities in Afghanistan (61%), Senegal (68.9%), and the DRC (77.2%) reported receiving funds from nongovernment sources.

**Table 1:** Percentage distribution of surveyed facilities according to their background characteristics in the 10 countries surveyed using the SPA.

Variables	AFG (n = 84)	BAN (n = 1303)	KEN (n = 567)	MAL (n = 810)	NAM (n = 357)	NEP (n = 899)	RWA (n = 382)	SEN (n = 334)	TAN (n = 933)	DRC (n = 1061)
**Mean/%** ^**1**^										
**Managing authority**										
**Private/NGO**	89.6	5.6	45.2	44.3	18.8	6.6	30.1	6.9	99.7	34.2
**Public**	10.4	94.4	54.8	55.8	81.2	93.4	69.9	93.1	0.30	65.8
**External sources of revenue**										
**None**	25.7	1.0	8.2	13.2	7.8	12.0	18.8	12.3	5.1	15.9
**Nongovt.**	61.0	5.1	6.6	26.7	31.4	7.0	11.8	68.9	25.3	77.2
**Govt.**	13.3	93.9	85.2	60.1	60.8	81.0	69.4	18.8	69.6	6.9
**Routine quality assurance activities performed**										
**No**	74.0	89.4	73.7	87.3	68.9	80.0	43.2	34.7	84.6	49.3
**Yes**	26.0	10.6	26.3	12.7	31.1	20.0	56.8	65.3	15.4	50.8
**Routine management meetings held**										
**No**	5.2	na	33.5	17.9	37.0	19.3	5.2	8.1	21.6	7.2
**Yes**	94.8	na	66.5	82.2	63.0	80.7	94.8	91.9	78.4	92.8
**External supervision conducted**										
**No**	27.2	9.8	12.9	18.4	25.8	37.0	6.3	9.9	8.1	0.90
**Yes**	72.8	90.2	87.1	81.6	74.2	63.0	93.7	90.1	91.9	99.1
**User fees**										
**No**	9.0	96.9	2.0	57.2	4.5	82.2	1.6	3.2	8.4	2.8
**Yes**	91.0	3.1	98.0	42.8	95.5	17.8	98.4	96.8	91.6	97.2
**Presence of health provider 24 hours/day**										
**No**	22.8	93.5	46.9	74.2	78.4	89.2	9.7	18.1	44.9	0.90
**Yes**	77.2	6.5	53.1	25.8	21.6	10.8	90.3	81.9	55.1	99.1
**Client feedback reviewed**										
**No**	61.9	71.7	60.6	53.9	51.8	55.0	90.6	61.8	55.2	52.0
**Yes**	38.1	28.3	39.2	46.1	48.2	45.0	9.4	38.2	44.8	48.0
**Number of FP providers**										
**Mean**	3.4	1.3	1.5	2.1	2.1	2.3	2.1	2.2	2.5	4.6
**Number of days FP services offered per month**										
**Mean**	24.4	21.0	20.3	16.5	17.7	23.8	19.4	22.6	20.5	25.2
**FP infection control measure score**										
**Mean**	4.7	3.8	4.1	4.2	4.1	3.7	3.7	6.6	4.3	3.8

Note: na = Data were not available.

^1^mean for continuous variables and the % for binary variables. Afghanistan (AFG), Bangladesh (BAN), Kenya (KEN), Malawi (MAL), Namibia (NAM), Nepal (NEP), Rwanda (RWA), Senegal (SEN), Tanzania (TAN), and the Democratic Republic of Congo (DRC)

Most health facilities in the countries studied did not have routine quality assurance systems in place, did not review client feedback, had external supervision, and had fixed user fees for all services. In the countries under assessment, FP services were provided on an average of 16.5 to 25.2 days per month, and there were on average 1.3 to 4.6 FP providers. From a low of 6.5% in Bangladesh to a high of 99.1% in the DRC, there were health professionals present 24 hours a day. Scores for FP infection control measures range from an average of 3.7 for Neal and Rwanda to 6.6 for Senegal in the countries being evaluated.

### Availability of FP services

Table 2 shows the country-by-country distribution for the availability of guidelines, equipment, and commodities among the surveyed facilities. The chi-square test showed that there were significant variations in all 17 indicators of the availability of guidelines, equipment, and commodities between the investigated countries. Senegal had the highest proportion of FP guidelines available (85.8%), whereas Afghanistan had the lowest (23.1%). Senegal also had the highest proportion of trained FP staff available (92.2%). BP apparatuses were widely available at the majority of facilities in all 10 countries studied, as were examination beds or couches.

**Table 2:** Percentage distribution of surveyed facilities according to the availability of guidelines, equipment, and commodities in the 10 countries surveyed using the SPA.

Variables	AFG (n = 84)	BAN (n = 1303)	KEN (n = 567)	MAL (n = 810)	NAM (n = 357)	NEP (n = 899)	RWA (n = 382)	SEN (n = 334)	TAN (n = 933)	DRC (n = 1061)	Chi-square statistic, *P*-value^5^
**%**											
**Staff and guidelines**											
**Presence of FP guidelines** ^**1**^	23.1	53.5	25.1	57.0	25.5	40.4	42.7	85.8	68.3	47.1	715.3, <0.001
**Availability of trained FP staff** ^**2**^	41.9	30.5	34.2	49.7	54.9	31.4	61.3	92.2	32.5	89.8	1448.8, <0.001
**Equipment and supplies**											
**BP apparatus** ^**3**^	97.0	86.1	87.6	70.1	91.0	87.1	87.4	82.2	79.4	87.9	188.3, <0.001
**Examination light**	68.8	46.8	43.6	27.8	35.9	45.2	14.9	69.4	13.0	55.1	728.9, <0.001
**Examination bed or couch**	97.9	76.2	96.7	86.8	94.4	82.7	85.1	79.4	87.1	75.8	234.0, <0.001
**Sample FP methods**	39.2	66.4	61.8	87.5	62.5	29.9	66.8	67.4	88.0	79.4	1043.5, <0.001
**Model for showing condom use**	2.1	36.2	31.9	45.3	56.9	10.8	50.3	22.4	42.4	32.0	488.4, <0.001
**Other FP- specific visual aid** ^**4**^	22.4	68.2	18.5	64.6	11.8	60.4	47.4	53.2	48.5	35.7	839.1, <0.001
**FP commodities provided**											
**Combined oral pill**	93.7	67.1	91.0	90.7	94.4	94.8	91.9	82.1	88.1	57.2	842.6, <0.001
**Progestin-only oral pill**	52.8	49.5	76.8	58.6	90.5	2.8	86.9	81.5	67.5	46.4	1664.9, <0.001
**Progestin-only injectable**	77.2	81.6	92.3	97.6	97.5	95.0	8.4	81.7	91.0	50.2	1172.2, <0.001
**Male condom**	79.3	95.0	89.4)	85.0	98.9	96.5	89.8	91.9	84.8	81.0	249.3, <0.001
**IUD**	76.9	33.2	55.2	17.5	5.6	21.3	14.9	66.2	27.2	31.6	545.4, <0.001
**Implant**	28.6	9.0	30.6	69.4	0.6	20.5	46.9	72.4	54.7	56.5	1549.4, <0.001
**Tubal ligation**	69.6	5.0	9.2	12.4	6.7	3.0	6.5	2.2	3.4	10.9	648.9, <0.001
**Vasectomy**	6.3	4.5	4.7	3.9	2.0	2.7	3.9	0.6	1.8	2.7	30.7, <0.001
**Emergency contraception**	31.4	14.5	72.1	62.4	29.1	10.4	14.4	79.4	22.4	30.0	1463.4, <0.001

Notes:

^1^ National guidelines or any other FP guidelines.

^2^ The facility had at least one interviewed staff member providing the service who reported receiving in-service training in some aspect of FP during the 24 months preceding the survey.

^3^A functioning digital BP apparatus or a manual sphygmomanometer with a stethoscope.

^4^ Flip charts or leaflets.

^5^ Chi-square test was performed.

Compared with the other countries, Afghanistan had the smallest proportion of sample FP methods (39.2%) and models for demonstrating condom use (2.1%). Namibia had the lowest proportion of other FP-specific visual aids (11.8%), such as flip charts or brochures, compared with the other countries. Tanzania’s healthcare facilities had the lowest proportion of examination lights (13%).

There were substantial differences among the countries in terms of the provision of modern FP methods. Except for Nepal and Rwanda, where progestin-only oral pills and progestin-only injectables were provided at 2.8% and 8.4% of the facilities, respectively, the most common methods provided by health facilities in all countries studied were combination oral pills, progestin-only oral pills, progestin-only injectables, and male condoms. In all countries except Nepal, tubal ligation and vasectomy were provided at a small proportion of facilities, with tubal ligation being done in roughly 70% of the facilities. Senegal’s health facilities provided more implants (72.4%) and emergency contraception (79.4%) than the other countries.

### Survey facilities’ readiness scores

The distribution of readiness scores to offer FP services per country is shown S2 Table. Senegal had the highest average readiness score, while Nepal had the lowest. Between the 10 countries, 4% to 36% of the facilities reported at least 75% (12–13/17) of the relevant items for FP service provision, with Senegal (36.1%) reporting the highest proportion and Nepal (3.6%) reporting the lowest.

S2a and S2b Fig. present histograms of readiness scores based on the 17 key items for the provision of FP in the countries studied. The majority of facilities in Afghanistan (55.9%) and Rwanda (67.3%) were clustered around 7 to 11 scores; the majority of facilities in Bangladesh (59.0%) and Nepal (61.3%) were clustered around 6 to 10 scores; the majority of facilities in Malawi (64.3%) and Tanzania (61.8%) were clustered around 8 to 12 scores; the majority of facilities in Kenya (60.9%) were clustered around 8 to 13 scores; the majority of facilities in Namibia (68.9%) were clustered around 10 to 12 scores; the majority of facilities in Senegal (64.5%) were clustered around 9 to 13 scores; and the majority of facilities in the DRC (55.2%) were clustered around 7 to 12 scores. According to the histograms, no facilities in Namibia, Rwanda, and Senegal had all 17 essential FP components.

### Factors associated with readiness to provide FP services

Table 3 presents the findings of the negative binomial regression model analysis for variables related to a health facility’s readiness to provide FP services in the countries studied. Assuming that the other variables in the model remained unchanged, the readiness scores were expected to rise for public- versus private/NGO- managed facilities in the following countries: Kenya, 10% (IRR 1.10; 95% CI 1.02 to 1.20); Malawi, 12% (IRR 1.12; 95% CI 1.04 to 1.20); Namibia, 12% (IRR 1.12; 95% CI 1.06 to 1.19); Nepal, 14% (IRR 1.14; 95% CI 1.00 to 1.29); Rwanda, 17% (IRR 1.17; 95% CI 1.07 to 1.27); and Tanzania, 14% (IRR 1.14; 95% CI 1.00 to 1.31).

**Table 3:** Models of negative binomial regression for variables associated with a health facility’s readiness to provide FP services in the 10 countries surveyed using the SPA.

Variable	AFG (n = 84)	BAN (n = 1303)	KEN (n = 567)	MAL (n = 810)	NAM (n = 357)	NEP (n = 899)	RWA (n = 382)	SEN (n = 334)	TAN (n = 933)	DRC (n = 1061)
**IRR (95% CI)** ^**1**^										
**Managing authority**										
**Private/NGO**	1.00	1.00	1.00	1.00	1.00	1.00	1.00	1.00	1.00	1.00
**Public**	1.16 (0.78–1.70)	0.86 (0.70–1.07)	**1.10 (1.02–1.20)** ^***c***^	**1.12 (1.04–1.20)** ^**b**^	**1.12 (1.06–1.19)** ^**b**^	**1.14 (1.0–1.29)** ^**c**^	**1.17 (1.07–1.27)** ^***a***^	1.09 (0.95–1.25)	**1.14 (1.00–1.31)** ^**c**^	1.03 (0.96–1.12)
**External sources of revenue**										
**None**	1.00	1.00	1.00	1.00	1.00	1.00	1.00	1.00	1.00	1.00
**Nongovt.**	0.91 (0.79–1.04)	0.87 (0.70–1.09)	1.11 (0.95–1.28)	0.98 (0.92–1.04)	1.01 (0.99–1.00)	0.97 (0.85–1.10)	0.96 (0.86–1.07)	**0.92 (0.86–0.98)** ^**b**^	1.03 (0.93–1.15)	0.98 (0.88–1.09)
**Govt.**	**0.65 (0.45–0.94*)*** ^**c**^	0.85 (0.71–1.02)	1.03 (0.92–1.15)	1.01 (0.95–1.07)	1.04 (0.93–1.09)	1.02 (0.95–1.10)	1.05 (0.97–1.15)	0.98 (0.92–1.05)	1.00 (0.90–1.11)	0.97 (0.83–1.15)
**Routine quality assurance activities performed**										
**No**	1.00	1.00	1.00	1.00	1.00	1.00	1.00	1.00	1.00	1.00
**Yes**	0.96 (0.84–1.09)	1.07 (0.98–1.17)	**1.13 (1.06–1.22)** ^**b**^	**1.09 (1.03–1.14)** ^**b**^	1.00 (0.96–1.04)	1.04 (0.99–1.10)	1.03 (0.97–1.09)	**1.16 (1.09–1.23)** ^**a**^	1.03 (0.96–1.10)	0.97 (0.90–1.05)
**Routine management meetings held**										
**No**	1.00		1.00	1.00	1.00	1.00	1.00	1.00	1.00	1.00
**Yes**	1.08 (0.84–1.41)	na	1.08 (0.99–1.19)	**1.10 (1.05–1.16)** ^**a**^	**1.03 (1.01–1.07)** ^**c**^	**1.08 (1.02–1.14)** ^**b**^	1.01 (0.93–1.11)	**1.15 (1.02–1.30)** ^**c**^	**1.06 (1.01–1.12)** ^**c**^	**1.23 (1.04–1.46*)*** ^***c***^
**External supervision conducted**										
**No**	1.00	1.00	1.00	1.00	1.00	1.00	1.00	1.00	1.00	1.00
**Yes**	0.89 (0.76–1.04)	1.03 (0.93–1.14)	**1.26 (1.10–1.45)** ^**b**^	**1.06 (1.01–1.11)** ^**c**^	1.02 (0.98–1.06)	0.99 (0.94–1.04)	1.13 (0.99–1.30)	1.00 (0.92–1.10)	0.97 (0.89–1.05)	1.93 (0.85–4.35)
**User fees**										
**No**	1.00	1.00	1.00	1.00	1.00	1.00	1.00	1.00	1.00	1.00
**Yes**	**0.58 (0.33–0.98)** ^**c**^	0.89 (0.71–1.13)	0.92 (0.83–1.03)	**1.08 (1.03–1.14)** ^**b**^	1.10 (0.98–1.31)	**1.15 (1.08–1.23)** ^**a**^	0.98 (0.80–1.20)	1.09 (0.88–1.36)	1.07 (0.97–1.17)	**0.83 (0.74–0.94)** ^**b**^
**Presence of health provider 24 hours/day**										
**No**	1.00	1.00	1.00	1.00	1.00	1.00	1.00	1.00	1.00	1.00
**Yes**	**1.45 (1.13–1.87)** ^**b**^	**1.14 (1.05–1.23)** ^**b**^	**1.18 (1.08–1.28)** ^**a**^	0.99 (0.95–1.03)	1.01 (0.97–1.06)	1.06 (0.98–1.14)	0.95 (0.85–1.06)	**1.20 (1.10–1.32)** ^**a**^	**1.07 (1.01–1.12)** ^***c***^	1.05 (0.77–1.41)
**Client feedback reviewed**										
**No**	1.00	1.00	1.00	1.00	1.00	1.00	1.00	1.00	1.00	1.00
**Yes**	0.96 (0.86–1.07)	**1.10 (1.03–1.17)** ^**b**^	0.95 (0.88–1.03)	1.03 (0.99–1.06)	0.98 (0.95–1.01)	**1.05 (1.00–1.10)** ^**c**^	0.89 (0.79–1.00)	1.04 (0.99–1.109)	1.01 (0.97–1.06)	1.00 (0.93–1.08)
**Number of FP providers**										
**Median (IQR)**	1.00 (0.99–1.01)	**1.08 (1.05–1.11)** ^**a**^	**1.07 (1.03–1.10)** ^**a**^	**1.05 (1.04–1.07)** ^**a**^	**1.14 (1.03–1.33)** ^**c**^	**1.07 (1.05–1.08)** ^**a**^	**1.06 (1.03–1.10)** ^**b**^	**1.04 (1.01–1.10)** ^**b**^	**1.05 (1.04–1.07)** ^**a**^	**1.03 (1.02–1.04)** ^**a**^
**Number of days FP services offered per month**										
**Median (IQR)**	1.00 (0.98–1.02)	**1.01 (1.0–1.02)** ^**b**^	1.00 (0.99–1.01)	1.00 (0.99–1.00)	0.99 (0.98–1.00)	0.99 (0.99–1.00)	1.00 (0.99–1.00)	0.99 (0.98–1.00)	1.00 (0.99–1.00)	**1.01 (1.00–1.01)** ^**b**^
**Median (IQR)**	**1.05 (1.00–1.09)** ^**c**^	**1.06 (1.05–1.08)** ^**a**^	**1.07 (1.04–1.11)** ^**a**^	**1.05 (1.04–1.07)** ^**a**^	**1.17 (1.05–1.33)** ^***b***^	**1.05 (1.03–1.06)** ^**a**^	**1.04 (1.01–1.06)** ^**a**^	**1.04 (1.01–1.07)** ^**b**^	**1.05 (1.04–1.06)** ^**a**^	**1.05 (1.03–1.08)** ^**a**^

Note: na = Data were not available.

^1^ CI = confidence interval; IRR = incidence rate ratios. Here a, b, and c indicate *p*<0.001, *p*<0.01 and *p*<0.05.

In Senegal, compared with facilities receiving no external funding, the readiness scores for those receiving nongovernmental funding were 8% lower, whereas in Afghanistan compared with facilities receiving no external funding the readiness scores was decreased by 35% when external sources of revenue were received from the government. In Kenya, Malawi, and Senegal, compared to those who did not, routine quality assurance activities were associated with increases in readiness of 13%, 9%, and 16%, respectively. Comparing holding routine management meetings to not holding them, readiness scores increased in Malawi (10%), Namibia (3%), Nepal (8%), Senegal (15%), Tanzania (6%), and the DRC (23%).

In Afghanistan, Bangladesh, Kenya, Senegal, and Tanzania, the presence of a trained healthcare provider 24 hours a day was associated with increases in readiness scores of 45%, 14%, 18%, 20%, and 7%, respectively, compared to the absence of such a presence. Fixed user fees were predicted to increase readiness by 8% in Malawi and 15% in Nepal. However, they were expected to decrease readiness by 42% and 17% in Afghanistan and the DRC, respectively. In Bangladesh and Nepal, health facilities that reviewed client feedback were associated with higher readiness scores than those that did not.

In all 10 countries, the readiness scores would increase if the number of FP service providers increased by one unit (per provider), as follows: 0.0004 (Afghanistan), 0.0008 (Bangladesh), 0.0007 (Kenya), 0.0005 (Malawi), 0.0014 (Namibia), 0.0007 (Nepal), 0.0006 (Rwanda), 0.0004 (Senegal), 0.0005 (Tanzania), and 0.0003 (DRC). In all countries studied, a higher FP infection control measure score was linked to higher readiness scores. In Bangladesh and the DRC, the number of days that FP services were offered each month was associated with higher readiness scores.

## Discussion

This was the first study to investigate the state of FP service readiness in 10 low-resource SA and SSA countries (Afghanistan, Bangladesh, Kenya, Malawi, Namibia, Nepal, Rwanda, Senegal. Tanzania, and the DRC) and the factors that influenced FP service readiness in those countries. We found four significant results. First, at least 75% (12–13/17) of the relevant items for FP service provision were found in 3% or more of the health facilities (Afghanistan: 15.8%; Bangladesh: 8.9%; Kenya: 14%; Malawi: 18.4%; Namibia: 26.3%; Nepal: 3.6%; Rwanda: 15.2%; Senegal: 34.1%; Tanzania: 13.7%; and the DRC: 15.5%). Second, no facilities in Namibia, Rwanda, and Senegal had all 17 essential items for FP service provision. Third, the countries varied significantly in the availability of all FP tracer items. Last, although the factors linked to increased readiness scores differed by country, increases in the number of FP providers and infection control measures for FP were linked to increased readiness scores in all 10 countries.

The low readiness of health facilities in the countries studied showed that the health systems in these low-resource settings faced significant problems providing FP services. Previous small-scale and national-level investigations in Bangladesh [22], Kenya [23], Mozambique [24], Pakistan [25], Tanzania [26], and Uganda [27] found that health facilities were not ready to deliver FP services. Our findings indicated that health facilities’ readiness to deliver FP services in these low-resource countries need to be strengthened to address the growing unmet need for FP.

Our study found that most health facilities in all countries studied were well equipped in terms of basic equipment for examination and monitoring FP use (BP apparatus, examination light, bed or couch, and sample FP methods). However, there were disparities in the availability of FP tracer items among the countries, according to the research. For example, comparing Afghanistan with other countries, the proportion of facilities having FP guidelines was exceptionally low, whereas Bangladesh stood out among the countries as having the lowest proportion of staff trained in FP. Moreover, FP guidelines were more likely to be found at health facilities than were FP-trained staff in Bangladesh, Malawi, Nepal, and Tanzania. This is concerning because even if guidelines were available at a facility in those countries, if personnel were not taught to follow them, they may not be followed.

Although short-acting contraceptive methods, such as male condoms, combined oral contraceptive pills, and progestin-only injectable contraceptives, were the methods that most available at the health facilities in our study, these same health facilities were not ready to provide long-acting and permanent methods, such as IUDs, vasectomy, and tubal ligation onsite. This finding was consistent with previous studies [22–27]. In all countries studied, only a small proportion (0.6% to 6.3%) of facilities provided vasectomy as one of the two types of permanent methods despite vasectomy being a very effective and safe contraceptive technique for couples who choose not to have children or to stop having children. As a whole, LMICs have a vasectomy rate of 2.5% [38, 39]. The lower prevalence of vasectomy in these countries may be partially due to the lack of availability of the procedure at healthcare facilities. As a result, enabling measures to expand service availability for this underutilized method are needed to improve or raise vasectomy uptake.

It is critical to maintain a sufficient number of health professionals to strike a balance between human and physical resources and to ensure a health system’s efficacy [40]. Because readily available healthcare consumables, such as drugs, supplies, and diagnostic instruments, have the potential to determine a healthcare system’s ability to recruit and retain healthcare specialists, and their ability to perform their job functions effectively, the link between an increase in the number of FP service providers and an increase in the readiness of health facilities to provide FP services is plausible in all 10 countries studied.

Likewise, we discovered that having trained health providers available 24 hours a day at the health facilities in Afghanistan, Bangladesh, Kenya, Senegal, and Tanzania was more likely to be associated with higher readiness scores. These findings are likely because this indicator was driving an expansion in the capability of certain health facilities to supply important items for FP service provision.

We found that increases in infection control measure readiness for FP exams were linked to higher overall readiness to provide FP services at the health facilities in all 10 countries studied. This is consistent with the finding from a descriptive exploratory study conducted in Botswana [41], which found that failure to comply with infection control measures was linked to a lack of necessary infrastructure, insufficient equipment and materials, insufficient personnel, and a lack of sustainable in-service education.

According to the current study, the readiness scores of publicly managed facilities in Kenya, Malawi, Namibia, Nepal, Rwanda, and Tanzania were higher than those of privately/NGO-managed facilities. These findings were consistent with earlier research done in low-resource settings [23, 26]. In contrast to private facilities, where the choice of services offered is based on the capacity of investment and the demand for services, the majority of public facilities in LMICs [17, 43]. do not have the mandate to choose the type of service to offer rather than to comply with government policies. This finding may be the result of this. Additionally, since commercial facilities typically charge for these services while public facilities provide FP for free, this may lower demand in the private sector and give the impression that it is not a viable investment. In order to obtain FP services, the data imply that the private sector’s capabilities must also be consistently used through formal public-private partnerships. According to research [17, 43]. factors limiting the ability of privately or NGO-managed facilities to offer FP services include dwindling money from donors, challenges in acquiring operating permits, and a lack of commitment on the part of the government in supporting the continuation or expansion of their activities. Therefore, it is important to pay attention to this.

Regular management meetings have been shown to improve a facility’s readiness for disease-specific [42] and FP process outcomes [43]. Regular meetings between facility managers and staff members improve a facility’s ability to provide appropriate services to clients. These meetings are occasionally used to assess whether health workers are correctly performing their duties. The results of our study indicated that routine management meetings were linked to higher readiness scores in Malawi, Namibia, Nepal, Senegal, Tanzania, and the DRC.

External supervision visits and routine quality assurance activities are important management methods for better integrating and improving service delivery at the health facility level [44, 45]. Our analysis found that routine quality assurance activities and external supervision were significantly associated with readiness scores in Kenya and Malawi, which was consistent with earlier research [24, 44, 45]. In Senegal, routine quality assurance procedures were associated with readiness scores.

Although there is currently conflicting research regarding how user fees influence people’s attendance at health facilities [46, 47], our research indicated that in Malawi and Nepal, a set user charge (for all services) was related to a higher readiness to deliver FP services. Health facilities in Malawi [48], and Nepal [49] had frequently shortages of recurrent inputs, such as medicines and other medical supplies, due to the lack of government investment. As a result, more cash from consumer fees for cost recovery might be used to improve healthcare quality, effectiveness, and coverage.

The current research has several strengths. First, the data were evaluated using the most recent samples of public and private health facilities in 10 SA and SSA countries, which included a substantial number of health facilities. Second, the SPAs employed rigorous interviewer preparation, standardized measurement instruments and methodologies, the same core questionnaire, and the pre-testing of tools to achieve standardization and comparability across multiple sites and timeframes. Third, the outcome variables were created using the SARA manual’s 10 measurements that reflected the clinical reality of the study. Fourth, we adjusted our findings for clustering effect and weighted the analysis to correct for non-response and unequal sampling by considering the complicated sampling methodologies involved. Last, based on the observations of professional survey enumerators, the presence or absence of protocols, equipment, and commodities at the health facilities were documented.

Several limitations apply to the findings presented here. Causation conclusions could not be drawn because the study used a cross-sectional sample. As a result, the current findings must be interpreted with caution. The availability of SPA surveys also placed limitations on the countries we could choose. Also, although there are SPA surveys for certain SSA nations, they are outdated (e.g., Zambia’s SPA survey from 2005) and do not include information on FP services (Uganda’s SPA survey from 2007). Because we only looked at SPA data from 10 SA and SSA countries, we cannot necessarily generalize the findings to other countries in the regions. We were unable to obtain data from FP clients, which would have provided further information from the perspective of service users; this was outside the scope of this research. Another limitation is that some countries conducted a census, some took samples of private facilities and a census of public facilities, and some took a sample of private and public facilities. Hence, the data are mixed. This might bias our findings.

Although the results of our study showed that regular management meetings were a significant factor in Malawi, Namibia, Nepal, Senegal, Tanzania, and the DRC having higher readiness scores, this information was not available for Bangladesh. Evidence has indicated that [13, 21]. hospitals tend to have substantially superior service readiness in terms of many factors when compared to health centers or clinics. As a result, it’s probable that the many factors considered for the FP readiness in our study will differ amongst the various health facility levels. However, since it is outside the purview of our study, we did not take this analysis into account. Future research may take this into account. Last, because there is no literature on the relative weight of each FP tracer item in the SARA instrument, all items in the FP-specific readiness scores were given equal weights. Therefore, the amount of FP service readiness may have been overestimated or inflated. Future research could contact FP healthcare specialists to increase the validity of FP readiness indicator (i.e., using Delphi methods).

### Conclusions

We found that health facility capacity to provide FP services was lacking in the 10 countries studied that have high unmet need for FP. Only a small proportion of the health facilities had at least 75% (12–13/17) of the FP items required for service provision. The countries differed greatly in the availability of all FP tracer items. Although the factors associated with higher readiness scores varied among the 10 countries, the number of FP providers and the presence of infection control measures for the FP exam were related to higher readiness scores. To increase a health facility’s readiness to offer FP services, country-specific factors must be addressed, in addition to the common factors found in all 10 countries. Further research is required to determine the cause of country-level differences in the availability of tracer items to develop targeted and effective country-specific strategies to improve service delivery preparedness in the SA and SSA regions.

## Supporting information

S1 Fig The sample selection of the facilities included in the SPA sample. (TIF)

S2 Fig a) Percentage distribution of surveyed facilities according to readiness scores in Afghanistan, Bangladesh, Kenya, Malawi, and Namibia. b) Percentage distribution of surveyed facilities according to readiness scores in Nepal, Rwanda, Senegal, Tanzania, and the DRC.
(ZIP)

S1 Table Survey details of 10 countries in the SA and SSA regions that took part in the SPA to assess service provision between 2007 and 2019. (DOCX)

S2 Table Overall distribution of readiness scores to provide FP services, by country. (DOCX)
