# Supplementary figures and images for: Assessing the readiness of health facilities to provide family planning services in low-resource settings: Insights from nationally representative service provision assessment surveys in 10 Countries
Source: PLoS One. 2023 Nov 16;18(11):e0290094. doi: 10.1371/journal.pone.0290094 (PMC10653533; doi:10.1371/journal.pone.0290094)

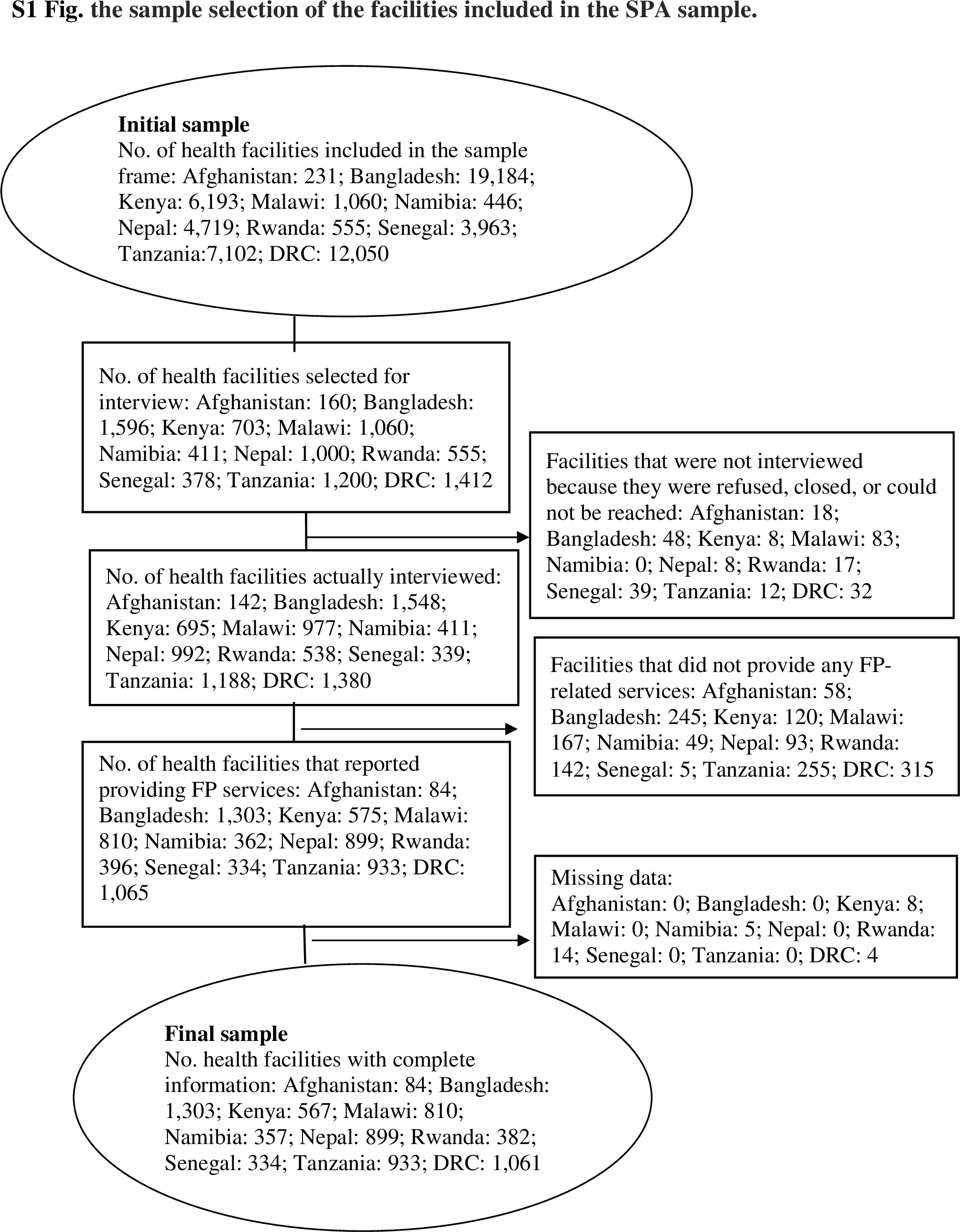

Supplement: S1 Fig — (TIF) [file pone.0290094.s001.tif]

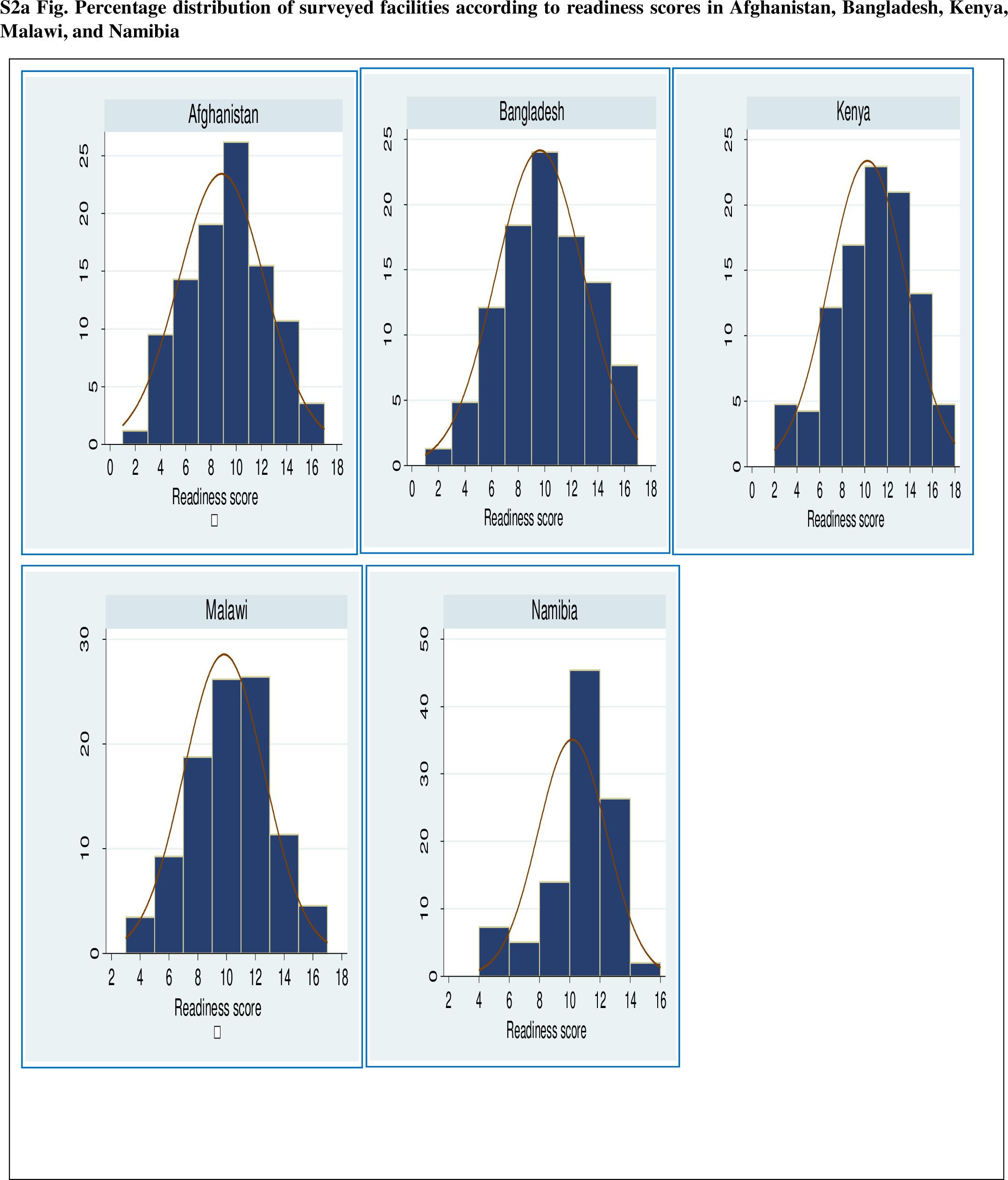

Supplement: S2 Fig — a) Percentage distribution of surveyed facilities according to readiness scores in Afghanistan, Bangladesh, Kenya, Malawi, and Namibia. b) Percentage distribution of surveyed facilities according to readiness scores in Nepal, Rwanda, Senegal, Tanzania, and the DRC. (ZIP) [file pone.0290094.s002.zip › S2a Fig.tif]

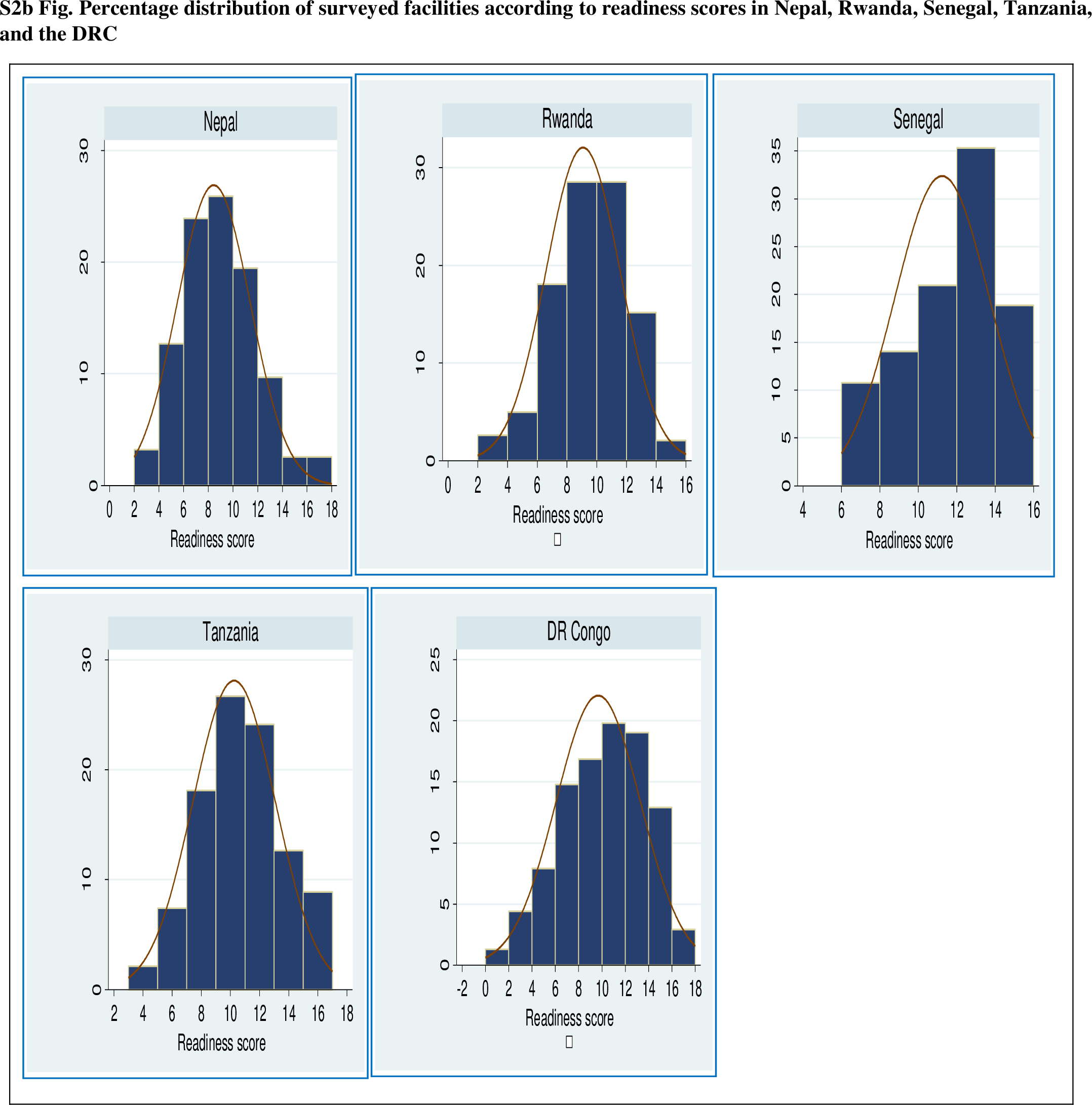

Supplement: S2 Fig — a) Percentage distribution of surveyed facilities according to readiness scores in Afghanistan, Bangladesh, Kenya, Malawi, and Namibia. b) Percentage distribution of surveyed facilities according to readiness scores in Nepal, Rwanda, Senegal, Tanzania, and the DRC. (ZIP) [file pone.0290094.s002.zip › S2b Fig.tif]
