# Supplementary material for: Assessing the readiness of health facilities to provide family planning services in low-resource settings: Insights from nationally representative service provision assessment surveys in 10 Countries
Source: PLoS One. 2023 Nov 16;18(11):e0290094. doi: 10.1371/journal.pone.0290094 (PMC10653533; doi:10.1371/journal.pone.0290094)
Supplement: S1 Table — (DOCX) [file pone.0290094.s003.docx]

**S1 Table** Survey details of 10 countries in the SA and SSA regions that took part in the SPA to assess service provision between 2007 and 2019

| Country | Survey year | Name of the survey | Facilities selected for the survey sample |
| --- | --- | --- | --- |
| Afghanistan | 2018–19 | Afghanistan Service Provision Assessment survey | Census of public hospitals. private hospitals and NGO/private clinics in six provinces namely Nangarhar, Paktya, Kunduz, Balkh, Kandahar, and Herat. In Kabul province there was a census of public hospitals. private hospitals and random selection of NGO/private clinics |
| Bangladesh | 2014 | Bangladesh Health Facility Survey | Combining a census of public hospitals (district hospitals and mother and child welfare centers) with random sampling of other public hospitals, private hospitals, and NGO/private clinics |
| Kenya | 2010 | Kenya Service Provision Assessment survey | Combining a census of public hospitals (national referral hospitals and provincial hospitals) with random sampling of other public hospitals, private hospitals, and NGO/private clinics |
| Malawi | 2013–14 | Malawi Service Provision Assessment survey | Census of public hospitals, private hospitals, and NGO/private clinics |
| Namibia | 2009 | Namibia Health Facility Census | Census of public hospitals, private hospitals, and NGO/private clinics |
| Nepal | 2015 | Nepal Health Facility Survey | Census of public hospitals, private hospitals, and NGO/private clinics |
| Rwanda | 2007 | Rwanda Service Provision Assessment survey | Combining a census of public hospitals with random sampling of private hospitals, and NGO/private clinics |
| Senegal | 2018 | Senegal Service Provision Assessment survey | Random sampling of public hospitals, private hospitals, and NGO/private clinics |
| Tanzania | 2014–15 | Tanzania Service Provision Assessment | Random sampling of public hospitals, private hospitals, and NGO/private clinics |
| Democratic Republic of the Congo | 2017–18 | Democratic Republic of the Congo Service Provision Assessment survey | Combining a census of public hospitals (tertiary level hospitals, provincial hospitals and general referral hospitals) with random sampling of other public hospitals, private hospitals, and NGO/private clinics |
