# Supplementary material for: Assessing the readiness of health facilities to provide family planning services in low-resource settings: Insights from nationally representative service provision assessment surveys in 10 Countries
Source: PLoS One. 2023 Nov 16;18(11):e0290094. doi: 10.1371/journal.pone.0290094 (PMC10653533; doi:10.1371/journal.pone.0290094)
Supplement: S2 Table — (DOCX) [file pone.0290094.s004.docx]

S2 Table Overall distribution of readiness scores to provide FP services, by country

| Country | Readiness scores |
| --- | --- |
|  | Mean ± SD |
| Afghanistan | 9.0 ± 3.1 |
| Bangladesh | 8.2 ± 3.0 |
| Kenya | 8.8 ± 3.0 |
| Malawi | 9.8 ± 2.8 |
| Namibia | 10.5 ± 1.7 |
| Nepal | 7.3 ± 2.4 |
| Rwanda | 9.0 ± 3.1 |
| Senegal | 11.1 ± 2.6 |
| Tanzania | 9.0 ± 2.5 |
| Democratic Republic of the Congo | 8.7 ± 3.5 |
